# Stakeholders’ experiences with the evidence aid website to support ‘real-time’ use of research evidence to inform decision-making in crisis zones: a user testing study

**DOI:** 10.1186/s12961-019-0498-y

**Published:** 2019-12-30

**Authors:** Ahmad Firas Khalid, John N. Lavis, Fadi El-Jardali, Meredith Vanstone

**Affiliations:** 1 Health Policy PhD Program, McMaster University, Hamilton, ON Canada; 2 Department of Health Research Methods, Evidence and Impact, McMaster University, Hamilton, ON Canada; 3 Centre for Health Economics and Policy Analysis, McMaster University, Hamilton, ON Canada; 4 McMaster Health Forum, McMaster University, Hamilton, ON Canada; 5 Department of Political Science, McMaster University, Hamilton, ON Canada; 6 Department of Health Management & Policy, American University of Beirut, Beirut, Lebanon; 7 Center for Systematic Review in Health Policy and Systems Research (SPARK), American University of Beirut, Beirut, Lebanon; 8 Knowledge to Policy (K2P) Center, American University of Beirut, Beirut, Lebanon; 9 Department of Family Medicine, McMaster University, Hamilton, ON Canada

**Keywords:** Evidence websites, evidence-informed decision-making, research evidence, crisis zones, evidence aid, user testing

## Abstract

**Background:**

Humanitarian action in crisis zones is fraught with many challenges, including lack of timely and accessible research evidence to inform decision-making about humanitarian interventions. Evidence websites have the potential to address this challenge. Evidence Aid is the only evidence website designed for crisis zones that focuses on providing research evidence in the form of systematic reviews. The objective of this study is to explore stakeholders’ views of Evidence Aid, contributing further to our understanding of the use of research evidence in decision-making in crisis zones.

**Methods:**

We designed a qualitative user-testing study to collect interview data from stakeholders about their impressions of Evidence Aid. Eligible stakeholders included those with and without previous experience of Evidence Aid. All participants were either currently working or have worked within the last year in a crisis zone. Participants were asked to perform the same user experience-related tasks and answer questions about this experience and their knowledge needs. Data were analysed using a deductive framework analysis approach drawing on Morville’s seven facets of the user experience — findability, usability, usefulness, desirability, accessibility, credibility and value.

**Results:**

A total of 31 interviews were completed with senior decision-makers (*n* = 8), advisors (*n* = 7), field managers (*n* = 7), analysts/researchers (*n* = 5) and healthcare providers (*n* = 4). Participant self-reported knowledge needs varied depending on their role. Overall, participants did not identify any ‘major’ problems (highest order) and identified only two ‘big’ problems (second highest order) with using the Evidence Aid website, namely the lack of a search engine on the home page and that some full-text articles linked to/from the site require a payment. Participants identified seven specific suggestions about how to improve Evidence Aid, many of which can also be applied to other evidence websites.

**Conclusions:**

Stakeholders in crisis zones found Evidence Aid to be useful, accessible and credible. However, they experienced some problems with the lack of a search engine on the home page and the requirement for payment for some full-text articles linked to/from the site.

## Background

Humanitarian action in crisis zones is fraught with many challenges, not the least of which is having rapid access to research evidence that has the potential to inform decisions. Acting on available research evidence can help to improve the effectiveness and efficiency of humanitarian interventions [1]. Access to research evidence to support decision-making is even more imperative in crisis zones because the magnitude and speed of the disaster creates a unique setting with known difficulties around accessing research evidence in a timely way (e.g. insufficient time, limited search skills, limited access to relevant evidence) [1–9]. Existing research has focused primarily on identifying the challenges decision-makers face in accessing evidence in crisis zones, highlighting the need for evidence websites to support evidence use in a timely way. However, because there has been so little research done on the experiences of stakeholders working in crisis zones with evidence websites, we currently do not know if such strategies address this key challenge. This analysis will help address this critical gap in the literature, contributing to efforts to support the use of research evidence in decision-making.

This gap persists in the existing literature for five main reasons. First, while literature exists that examines evidence websites in other settings, these studies do not focus on evidence use in crisis zones [10–12]. Second, user-testing studies have tended to focus on facets of user experience without first investigating the information needs of users [12–14]. This means that they potentially missed gaining valuable insight into how evidence websites can best meet stakeholders’ knowledge needs. Third, some studies have contributed evidence about best practices in organizing content, but there are many other facets of user experience that remain unexplored [15]. Fourth, studies have not explored stakeholders’ views of and experiences with using a database to find evidence summaries on specific health policy and systems-relevant questions [11, 15]. Finally, there is a lack of third-party research about the effectiveness of evidence websites, with most existing research designed and conducted by groups associated with the website under study [11, 12, 15].

In light of the lack of third-party research in this area, this study presents a non-affiliated examination of the use of Evidence Aid by a diverse array of stakeholders working in crisis zones. Evidence Aid is the only evidence website designed for crisis zones that focuses on providing research evidence in the form of systematic reviews. Systematic reviews critically appraise and summarize all relevant individual studies, which reduces the amount of time and search skills other stakeholders need to access and appraise large bodies of research [16]. Evidence Aid has invested efforts to improve the site, but such efforts have not yet been formally evaluated.

## Methods

### Study aim

Employing a user-testing study design, our objective herein was to explore the information needs of stakeholders working in crisis zones and their views of and experiences with the Evidence Aid website. This paper also aims to put forward specific suggestions about how to improve evidence websites designed to support the use of research evidence in decision-making in crisis zones. Many of these suggestions can also be applied to other evidence websites that support the use of evidence in decision-making more broadly.

### Study design

A user-testing study design was used to address our research objective. This type of design is widely employed in the field of product design and evaluation, and involves having users complete task-specific problems [17–19]. User testing involves inviting representative users of a product (in this case a website) to participate in individual semi-structured interviews where they are asked about their experience as they interact with the website [20]. This study used qualitative methods (e.g. interview data, thematic analysis, etc.) to describe users’ knowledge needs, views and experiences with using Evidence Aid, gathering specific suggestions about how their experiences could be improved. Qualitative research methods have the potential to drive improvements to the experience of using particular resources, creating information to allow developers to make user-centred improvements. Our use of a concurrent think-aloud protocol allowed us to access user thoughts in-the-moment, lessening the likelihood that users would forget their insights or dismiss them as unimportant when asked to discuss their experience at a later date [21, 22].

We started our interview with a set of preliminary general questions about the participant’s profession and knowledge needs followed by a set of think-aloud user experiences and views while performing task-specific questions. Our lack of involvement with Evidence Aid makes us particularly well positioned to elicit frank feedback. Participants were informed of our lack of involvement with Evidence Aid at the outset of the interview.

### Defining Evidence Aid

Evidence Aid (https://www.evidenceaid.org) is an English-language interface with some articles and user-friendly summaries available in Spanish and French. To be included in Evidence Aid, the systematic review must focus on the effectiveness of humanitarian action and include health-related outcomes. Evidence Aid provides appraisal for each of the systematic reviews. Research evidence is available on Evidence Aid in three ways — first, through a simple search bar located under a resources tab with the option of selecting month (e.g. March 2019) and category (e.g. emergency type); second, research evidence is organized into four main categories, namely health issues (i.e. burns, cardiovascular disease), emergency type (i.e. flood, epidemic), humanitarian cluster (i.e. camp coordination and camp management, emergency shelter) and person groups (i.e. adolescents, adults); finally, Evidence Aid produces curated collections of evidence specific to crisis zones (e.g. acute malnutrition, prevention and treatment in emergencies and humanitarian crises).

Evidence Aid provides free access through their website to some of the full-text articles available on other websites that usually require a payment (e.g. the Cochrane Library collection for earthquakes). However, some of the full-text articles available through the site do require a payment to access the content, although this is arguably outside of Evidence Aid’s scope given the nearly limitless liability they would face if they offered free access to all articles.

### Characteristics of participants

We purposively sampled two types of participants for the study — participants who have used Evidence Aid before and those who have not. Purposeful sampling allowed us to gain valuable insights and an in-depth understanding of stakeholders’ views and experiences with Evidence Aid to support evidence use in crisis zones [23]. All participants enrolled in the study were either currently working or have worked within the last year in a crisis zone. Participants were asked to self-identify the type of stakeholder they are based on their profession (e.g. senior decision-maker, advisor). We define stakeholder as “*anyone that has an interest in, is likely to be affected by, or has the ability to influence*” a decision ([24], p. 1939). All participants were asked the same general questions and user experience-related task questions. Those who have used Evidence Aid before were asked about how frequently they used the site, and this additional information was used to explore patterns in their views of and experiences with evidence websites in addressing their research evidence needs.

### Participant recruitment and sample size

Decision-making processes are complex and require a network of stakeholders with different types of expertise. The types of stakeholders involved in decision-making processes include advisors, analysts and researchers providing formal support to senior decision-makers, field managers and healthcare providers [25]. A two-stage sampling approach was used to identify and recruit key stakeholders [26, 27]. The first stage included identifying stakeholders in the following five categories based on their anticipated roles in decision-making in crisis zones and, where appropriate, across the humanitarian aid, health system and health research system sectors: (1) senior decision-makers (e.g. presidents, directors); (2) field managers (e.g. field coordinators, heads of missions) directly involved in coordination and management of crisis zones; (3) healthcare providers (e.g. doctors, nurses) involved with either the development of medical guidelines in crisis zones or directly delivering medical care to people in crisis zones; (4) advisors directly involved in advising about policy development and implementation strategies; and (5) analysts and researchers directly involved in responding to research evidence requests from the previous four categories of participants. The second stage of recruitment used snowball sampling; research participants in the first stage were asked to identify any additional potential stakeholders.

To capture users who have used Evidence Aid, we sent a LinkedIn email invitation to a list of 789 members who are part of a LinkedIn thematic working group named ‘Health Systems in Fragile and Conflict Affected States’. This thematic working group contained key actors in health who are working or have formerly worked in fragile and conflict-affected states and who were invited to participate in the Humanitarian Evidence Week initiative led by Evidence Aid on November 6–12, 2017. Participants who have not used Evidence Aid before were recruited in three ways. First, we included, in the same LinkedIn email invitation described above, a request to nominate colleagues who are in similar roles but who did not participate in Humanitarian Evidence Week and who did not use Evidence Aid. Second, we sent email invitations to those listed on a publicly available contact list for a quality improvement exercise conducted at Médecins Sans Frontières that focused on the organization’s approach in transferring research knowledge to policy and practice during the Syrian Refugee Crisis. Third, we sent email invitations to those identified through documentary and media analysis (using publicly available documents only).

We aimed at completing at least five user test interviews for each type of participant category (i.e. senior decision-makers, field managers, healthcare providers, advisors, analysts and researchers) for both types of participants (i.e. those that have used Evidence Aid and those that have not), recognizing that this estimate was dependent on the availability of appropriate participants. We recruited 9 participants from our first stage of sampling, and 22 additional participants were identified through snowball sampling. Our sample size amounted to a total of 31 participants (Table 1). Previous user testing studies highlighted that 80% of known usability problems could be obtained from 5 representative users, with diminishing returns after the fifth user [28].

**Table 1:** Profiles of respondents involved in the user-testing exercises

Type of stakeholder	% (*n*)	Organizational affiliations	Organizational types	Used Evidence Aid before	Sex
Senior decision-maker	26% (*n* = 8)	Médecins Sans Frontières (*n* = 4)	NGO (*n =* 6)	5 No	6 W
		International Federation of Red Cross and Red Crescent Societies (*n =* 2)	Government agency (*n* = 2)	3 Yes	2 M
		Palestinian National Institute of Public Health (*n =* 1)			
		Public Health England (*n =* 1)			
Advisor	23% (*n* = 7)	International Federation of Red Cross and Red Crescent Societies (*n* = 2)	NGO (*n* = 4)	5 No	4 W
		Pan American Health Organization (*n* = 2)	International agency (*n* = 3)	2 Yes	3 M
		Goal Global (*n* = 1)			
		United Nations High Commissioner for Refugees (*n* = 1)			
		World Vision Canada (*n* = 1)			
Field manager	23% (*n* = 7)	International Federation of Red Cross and Red Crescent Societies (*n = 2)*	NGO (*n = 7)*	4 No	4 W
		International Rescue Committee *(n = 2)*		3 Yes	3 M
		International Medical Corps (*n =* 1)			
		Médecins Sans Frontières (*n =* 1)			
		Save the Children Canada (*n =* 1)			
Analyst/researcher	16% (*n* = 5)	ALNAP (*n* = 1)	NGO (*n* = 3)	4 No	3 W
		International Federation of Red Cross and Red Crescent Societies (*n* = 1)	Academic institution (*n* = 1)	1 Yes	2 M
		Médecins Sans Frontières (*n* = 1)	International agency (*n* = 1)		
		University of Amsterdam (*n* = 1)			
		World Health Organization (*n* = 1)			
Healthcare provider	13% (*n* = 4)	Médecins Sans Frontières (*n* = 3)	NGO (*n* = 4)	4 No	3 W
		Rose Charities and Association of Medical Doctors of			1 M
		Asia (*n* = 1)			

^*M* men; *NGO* non-governmental organization; *W* women^

### Data collection methods

Interviews were conducted via Skype by the first author (AFK), who acted as both the interviewer and note taker. The interviews lasted approximately 60 minutes and were audio-recorded after receiving permission from the participant. Audio recordings were transcribed verbatim and the written transcriptions were used for data analysis. Potentially identifying information (e.g. name) was removed at the time of transcription. We conducted the interviews in English, which is the language used in the Evidence Aid interface.

The user testing began with preliminary questions about the participant’s profession, what sources of research evidence they use and knowledge of evidence websites including Evidence Aid (see Additional file 1 for more details). We provided participants with a set of instructions, starting from an empty browser window. This was followed by a series of tasks for the participant to perform, some of which involved looking for specific content tailored to their field or professional interests. For example, a healthcare provider in a crisis zone may choose to find a specific review about the effect of antibiotic resistance among children in refugee camps. Other general tasks asked of the participants included finding help, finding the search engine within Evidence Aid website and finding information about Evidence Aid. The concurrent think-aloud method was used throughout [19]. Additionally, participants were asked to state the major problems they faced, whether these were ‘big’ problems or frustrations while performing the task or minor issues, any positive feedback they would like to provide, and suggestions for improving their experience. We explained to participants that major problems are ones that have serious potential for causing erroneous usage of Evidence Aid and therefore unable to complete the intended tasks. Big problems are ones where users face frustration and difficulty in completing tasks but are able to work around the problem, and minor issues are those that slow down or inconvenience users unnecessarily in completing tasks [29–31]. Finally, to assess their overall experience with Evidence Aid, we asked questions related to Morville’s seven facets of the user experience — findability, usability, usefulness, desirability, accessibility, credibility and value [32].

### Data analysis

We used a deductive framework analysis approach towards our collected data [33, 34]. Framework analysis is a qualitative method that can be applied to research that has specific questions, professional participants and a limited time frame [34]. This approach allowed us to describe and interpret what is happening in a particular setting (i.e. use of Evidence Aid) by asking our participants specific questions [33]. It involved a five-step process that included familiarization (i.e. immersing ourselves in collected data making notes of key ideas and recurrent themes), identifying a thematic framework (i.e. recognizing emerging themes), indexing (i.e. using NVivo to identify sections of data that correspond to particular themes), charting (i.e. arranging identified sections of data into table exhibits), and mapping and interpretation (i.e. analysing key characteristics from the exhibits) [33].

Data were analysed by drawing on Morville’s seven facets of the user experience, as described above [32]. A detailed description of the seven facets of the user experience is provided in Table 3. Morville’s framework was selected because it combines the main facets of usability, incorporates the emotional aspects of user experience, and is often used in other user-testing studies to explore user experience in an information design context, which Morville refers to as the ‘honeycomb’ [12, 13, 35].

## Results

### Participant profiles

A total of 31 interviews were completed (Table 1), with senior decision-makers (*n* = 8), advisors (*n* = 7), field managers (*n* = 7), analysts/researchers (*n* = 5) and healthcare providers (*n* = 4). Good balance was achieved across types of organizations (e.g. non-governmental organizations, international agencies, government agencies and academic institutions). A high proportion of interviewees had not used Evidence Aid before (*n* = 22); 65% of the participants were women (*n* = 20) and 35% were men (*n* = 11); 17 interviewees have never heard of Evidence Aid before our interview, while 14 participants had heard and used Evidence Aid occasionally.

### Participant knowledge needs, types of information used to address knowledge needs and sources for obtaining information

Many of our participants highlighted the scarcity of available knowledge relevant to crisis zones, with one senior decision-maker stating:

“*There is never enough knowledge and evidence in fast evolving crisis, especially when we deal with emergencies and we never know what is going on and we are always desperate to get more information. The lack of ability to get … information during a fast-moving developing disaster situation is a massive challenge.*”

The distribution of participant knowledge needs, types of information used and sources for obtaining information varied depending on the type of stakeholder (Table 2). The following knowledge needs were most cited by a specific type of stakeholder: policy development related to health-system strengthening and health-advocacy approaches by senior decision-makers; operational logistical management (e.g. setting up mobile health clinics in crisis zones) by field managers; clinical management of patients in a crisis zones by healthcare providers; and community-level programme development (e.g. how to support behaviour change in a community setting) and implementation strategies for any of the above four domains cited by advisors and senior decision-makers, respectively.

**Table 2:** Users’ knowledge needs, types of information used, and sources for obtaining information^a^

Categories^a^	Senior decision-maker	Field manager	Healthcare provider	Advisor	Analyst/researcher
Knowledge needs					
1. Policy development (e.g., health-system strengthening, health-advocacy approach, etc.)	**✔**	✓	✓	✓	✓
2. Operational logistical management (e.g., military and political context, shelter, security, hygiene, mobile clinic set ups, human resources issues, cross-border health supplies management, etc.)	✓	**✔**		✓	✓
3. Clinical management of patients in a crisis situation (e.g., case management, etc.)		✓	**✔**	✓	✓
4. Community-level program development (e.g., behavior change support, etc.)				**✔**	
5. Implementation strategies for any of the above (i.e., policy development, operational logistical management, clinical management, and community level program development)	**✔**				
Types of information used^b^					
• Data:					
1. country specific registries and surveillance data	**✔**				
• Research evidence:					
1. *systematic reviews and meta-analyses*	✓	✓	✓	✓	**✔**
2. single context-specific case studies	✓	**✔**	**✔**	**✔**	✓
3. *intervention studies (e.g., clinical trials)*	**✔**		**✔**	**✔**	✓
4. surveys	✓	**✔**	✓	✓	
5. observational studies		**✔**			**✔**
6. conceptual papers (e.g., theoretical papers)		**✔**	**✔**		
• Guidance:					
1. internal guidance documents		**✔**	**✔**	✓	
2. *global guidelines (e.g., WHO)*	✓			**✔**	✓
• Expert opinion:					
1. expert opinions from the field		**✔**	**✔**	**✔**	**✔**
• Stakeholder insights:					
1. stakeholder tacit knowledge or ordinary knowledge		**✔**			✓
2. stakeholder opinions	**✔**	**✔**			
• Undefined combinations of the above:					
1. internal organizational field assessment information	✓	**✔**		✓	✓
2. lessons learned discussion papers					**✔**
Sources for obtaining information					
• Databases:					
1. one-stop shops:					
1. ReliefWeb: contains many different types of information but predominantly news and not research evidence	**✔**	✓	✓	✓	✓
2. Health Systems Evidence: contains systematic reviews on a given topic related to health-system arrangements or implementation strategies				**✔**	
3. Zika communication Network (ZCN): contains evidence-based toolkits and guidance related to Zika virus				**✔**	
2. organizational databases that provide only that organization reports (e.g., ACAPS, ALNAP^c^, Chatham house library, Cochrane)	✓	✓		**✔**	✓
3. organizational databases that provide access to other information (e.g., MEDBOX, WHO: HINARI)	✓	✓	✓	**✔**	✓
4. Google (e.g., google scholar, general google search)		✓	**✔**	**✔**	✓
5. indexed bibliographic databases accessed through University library subscriptions (e.g., Science Direct, Scopus, Up-to-date)		✓	✓	**✔**	**✔**
6. indexed bibliographic databases accessed through other mechanisms (e.g., PubMed)	✓	**✔**		✓	✓
7. organizational internal databases	**✔**	✓	**✔**	✓	
8. media websites (e.g., print media, broadcast media)	**✔**				
• Reports:					
1. reports by UN agencies (e.g., IOM, OCHA, UNHCR, UNICEF, WHO)	**✔**	✓	✓	**✔**	✓
2. internal reports (e.g., ICRC, MSF, ODI, Save the Children,)	✓	✓	**✔**	**✔**	**✔**
3. reports by US agencies (e.g., CDC, CIA fact sheets, USAID)	**✔**		✓	✓	
4. field staff reports	**✔**	✓			
5. reports by charitable organizations (e.g., Bill & Melinda Gates, Oxfam)		**✔**			
6. reports by UK agencies (e.g., Rebuild consortium)				**✔**	
• Correspondence and social networks:					
1. Email subscriptions (e.g., Disaster management information, Global Health Network, William Brighter Institute)	**✔**		**✔**	**✔**	
2. Social networking sites (e.g., Facebook, ResearchGate, Twitter)				✓	**✔**
3. memos and correspondence distributed across the whole organization					**✔**
4. direct correspondence with senior-decision makers	**✔**				
5. direct correspondence with review article authors	**✔**				

Bolded checkmarks **✔** indicate most cited by respondents. Multiple bolded checkmarks for the same category indicates equal frequency of citation by respondents

^**a**^Sub-categories are listed from the most cited to the least cited

^b^Italicized bullet points are within Evidence Aid scope (i.e., able/appropriate for EA to do given mission of website: “to alleviate suffering and save lives by providing the best available evidence on the effectiveness of humanitarian action and enabling its use”

^c^In addition to carrying out original research, ALNAP hosts a library of evaluations of humanitarian action from other sources

As for the types of information used by our participants to address their knowledge needs, we focus our attention here on those that are within Evidence Aid’s scope — systematic reviews and meta-analyses were most cited by analysts and researchers, while intervention studies (e.g. clinical trials) were most cited by senior decision-makers, healthcare providers and advisors. Global guidelines (e.g. from WHO) were most cited by advisors. Finally, our participants obtained information from a wide variety of sources (e.g. evidence websites such as ReliefWeb and Health Systems Evidence, reports by UN agencies, correspondence with senior decision-makers, and social networking sites such as Facebook and Twitter).

### User experiences

Overall, there were two notable differences in responses across our diverse types of stakeholders and between users and non-users of Evidence Aid. First, analysts and researchers we interviewed demonstrated enthusiasm that Evidence Aid is attempting to bring research evidence closer to humanitarian aid workers, while some senior decision-makers were sceptical about using Evidence Aid as opposed to relying on information stemming from their ground operations to answer specific questions. Additionally, participants that have used Evidence Aid before were more familiar with the organization of tabs on the website, which facilitated faster access to desired content than non-users. Finally, there were no notable differences in responses across gender.

Participants did not identify any ‘major’ problems (highest order) across the seven domains of the user experience (Table 3). However, participants identified two ‘big’ problems (second highest order) related to findability and accessibility. In terms of findability, participants frequently cited the lack of a search engine on the home page as a problem in locating desired articles. Turning to accessibility, participants expressed frustration that some of the full-text articles available through the site required a payment to access the content and that timely assessment data on current crisis is missing; provision of access to pay-walled research and timely assessment data is outside of the scope of Evidence Aid’s services. We outline below, by domain, the most frequently cited minor issues, positive feedback and specific suggestions.

**Table 3:** Users’ experiences using Evidence Aid^a^

Domain	Issues raised			Positive feedback	Specific suggestions
	Major problems	Big problems or frustrations	Minor issues		
Findability • Easy to find the site with a Google search or URL • Easy to locate desired articles using a search engine, tabs, closed dictionary or a combination • Easy to locate desired information about/from articles using initial display and/or supplementary webpages on site or on other sites	• None	• Home page does not have a search engine * • **Difficulty identifying correct terms to enter into the search engine**	• Difficulty locating the search bar * • **Difficulty locating user-friendly summaries** • **Lack of search categories to narrow down the search results** • **Latest content banner on home page is heavily focused on internal Evidence Aid activities and less on finding and retrieving resources to support evidence-informed decision-making in crisis zones** • **Difficulty locating supplemental information on other sites (e.g., dead links)**	• Four cluster areas under “Resources” are helpful in finding research evidence * • Results ‘tags’ further narrows the search results * • Website is easy to find with a Google search or URL • Clearly marked tabs on home page to arrive at Resources, Events, etc., • Search bar under Resources is helpful • Rapid display of search results	• Add an advanced search filter (e.g., date of last search, specific contexts, language preference) for more targeted search results * • **Bring forward to the Home page the following:** **▪ search engine** **▪ four cluster areas** **▪ feature systematic reviews and best practice health guidelines related to a current crisis** **▪ organize search results according to target user (e.g., researcher, decision-maker, etc.)**
Usability • Purpose and scope of the site clearly described • Basic tasks easily accomplished on first use • Search goals achieved with effectiveness, efficiency and satisfaction	• None	• None	• Basic tasks require undertaking multiple steps to arrive at results on first use * • **Not clear what the purpose of the website is, target audience, or the type of evidence it provides** • **Not clear how Evidence Collection is created and how it is different from the available research evidence on website**		• Create a clearer statement on purpose of site and type of evidence it provides * • **Promote and clarify the purpose of Evidence Collection**
Usefulness • Nature of information retrieved provides value (e.g., addresses question without jargon and in an understood language)	• None	• None	• **Little focus on current and ongoing crises – most evidence presented is post crisis** • **User-friendly summaries do not contain enough relevant details to make an informed decision on whether to read the full article** • Lack of systematic reviews and guidelines related to: ❖ participants’ particular areas of professional interests or field of work * ❖ context-specific research evidence ❖ all answers to questions relevant to humanitarian action ❖ site, search results, user-friendly summaries, and full text in different languages • Lack of single studies (e.g., “real-time” data and evaluations) in addition to systematic reviews and guidelines	• Useful in providing an independent evidence website for curated evidence on crisis zones for decision-makers working in the field * • Once located: ❖ user-friendly summary is concise, easy to understand, and practical in deciding whether to read full text ❖ the evidence-based guidelines are good in providing take-home messages • Contains evidence related to both man-made and natural disasters • Provision of some content on the site in other languages (e.g., Spanish) is useful • Contains more systematic reviews than other websites • Fills the gap between academic research (i.e., systematic reviews) and action in humanitarian aid settings • Resources tab regarding recent crises is important	• Better linkage of evidence into action – turning the evidence available into explicit actionable points for decision makers in crisis zones * • **Include research evidence that addresses:** **▪ strengthening health systems** **▪ implementation strategies for interventions in humanitarian crisis** **▪ building capacity towards use of research evidence** • Add more content in other languages • Survey end-users about their needs
Desirability • Images reflect the purpose and scope • Design conveys a unique and appropriate identity (e.g., name, logo, font type & size, colours, and sophistication of features)	• None	• None	• Photos are ordinary (i.e., academic looking) and repetitive * • **Amount of white background on screen is problematic for people who work on big screens & high definition** • **Font size is smaller than some respondents would prefer** • **Logo is similar to the ONE and MSF website**	• Basic design: simple, not a lot of pop ups * • Home page is clean and organized • Good choice of colors on the website • “Evidence Matters” is catchy • Title: “Evidence Aid” is appealing	• Use compelling photos relevant to humanitarian contexts * • **Use infographics to breakdown key findings from the evidence**
Accessibility • Accommodates diverse user contexts (e.g., inability to pay article-access fees or avoid them through affiliations with universities with paid subscriptions, inability to use high bandwidth features) • Accommodates diverse physical functioning in the user (e.g., colour choices accommodate color blindness, font size is changeable)	• None	• Some of the evidence available is not open-access (i.e., requires a payment) *	• Widespread use of the color red creates challenges for those with color blindness * • Concerns over whether documents can be read online or have to be downloaded first - a problem in a low bandwidth internet setting *	• Accessible to a broad spectrum of people working in the humanitarian sector who have access to the internet * • Useful during a current large-scale humanitarian crisis or for select other topics in providing time-limited free access to full-text articles that are normally behind a pay wall	• Create a mobile friendly app or use responsive design *
Credibility^a^ • Easy to verify accuracy of information on site (e.g., clear indication of inclusion criteria of research evidence, objective assessment of available research evidence, links to credible external sources (e.g., Cochrane), provides complete exhaustive summary of evidence (e.g., systematic reviews)) • Clear illustration that honest and trustworthy people stand behind the site (e.g., profile description of people) • Site is updated frequently (i.e., content been reviewed recently) • Restraint in any promotional content (e.g., ads) or direct link to a particular funding source	• None	• None	• Not clear what inclusion criteria is used to include the best available evidence * • Concerns over the frequency of updating latest available research evidence	• Direct and clear link to Cochrane Library increased level of trust by respondents * • Seems to be continuously updated with the newest content upfront for easier access • Team and advisory committee behind the site are credible individuals • Objective assessment of research evidence • Availability of systematic reviews • Available research evidence does not seem to be directly linked to a particular funding source	• Give greater visibility to major contributors & funders *
Value for the user • Intended users are aware of the site • The site advances the mission of the organization: “to alleviate suffering and save lives by providing the best available evidence on the effectiveness of humanitarian action and enabling its use”	• None	• None	• Lack of awareness among humanitarian aid workers about the existence of or value added by Evidence Aid *	• A solid attempt to putting together multiple sources of information in one spot * • Substantial efforts in partnering with other organizations to fill gaps with new systematic reviews • Specificity to humanitarian action is helpful	• Emphasize why evidence matters in humanitarian action * • Continue collaborating with other organizations to fill gaps with new systematic reviews * • **Choose from a menu of additional ways to enable the use of the site and its contents (e.g., personalized briefing notes, rapid evidence synthesis, webinars, etc.)**

* bullet points indicate most cited by respondents. Multiple * points for the same category indicates equal frequency of citation by respondents

Bolded bullet points are within Evidence Aid scope

Positive feedback column does not use bolded bullet points to indicate whether the respondent’s feedback is within Evidence Aid scope

^a^We adapted Stanford University guidelines for web credibility, based on three years of research that included over 4,500 views and experiences, to assess the extent to which interviewed participants trust and believe what is presented to them and what elements of Evidence Aid influenced this trust [53]

### Findability

Participants cited a minor issue of having difficulty locating the search bar. As for positive feedback, participants indicated that the four cluster areas (i.e. health issues, emergency type, humanitarian cluster and person groups) under the ‘Resources’ tab were helpful in locating desired information. In addition, participants appreciated that the ‘tags’ in the results page helped to further narrow down their search results. Participants suggested the addition of an advanced search filter for more targeted search results (e.g. date of last search, specific contexts and language preference).

### Usability

Participants cited as a minor issue having to undertake multiple steps to perform basic tasks to arrive at results on first use. However, some participants did note that, once they had enough time on the site, they were able to perform basic tasks efficiently. A field manager commented:

“*I appreciate that there is a learning curve until one is familiar with the site and how to use it efficiently.*”

To improve the usability of the site, some participants suggested creating a clearer statement of the site’s purpose and the type of evidence it provides.

### Usefulness

For minor issues, participants sometimes cited a lack of systematic reviews and guidelines related to their own particular areas of professional interests or fields of work. Participants provided positive feedback related to how useful the site is in providing an independent evidence website for curated evidence on crisis zones for decision-makers working in the field. As one senior decision-maker commented:

“*It is good for humanitarian workers to have all the articles on one site so they can go there and look for evidence-based approaches.*”

Most participants suggested that Evidence Aid should focus some of their efforts on turning the evidence available into explicit actionable points for decision-makers to use in crisis zones. A stakeholder highlighted this suggestion by stating:

“*Most people in the humanitarian sector do not understand abstracts and they almost alienate them. A better strategy is friendly-summary reviews that are shorter, to the point, with clear actionable points.*”

### Desirability

Participants cited a minor issue of photos on Evidence Aid being ‘ordinary’ (i.e. academic looking) and repetitive. A healthcare provider explained Evidence Aid choice of pictures on the home page stating:

“*Photos make it seem like a training workshop website with the pictures of classroom settings.*”

Photos displayed on Evidence Aid prompted many participants (including the above healthcare provider) to suggest that the developers behind the site should consider using compelling photos that are relevant to humanitarian contexts. Participants did appreciate the basic simple design of the site and the lack of numerous pop-up advertisements.

### Accessibility

Participants cited concerns over whether documents can be read online or have to be downloaded first, the latter of which can be a problem in a low-bandwidth internet setting and would pose a significant limitation to those using the site from the frontlines of a crisis zone, with a healthcare provider stating:

“*Access to the internet in the field is a big barrier. It is a touch and go situation.*” – healthcare provider working in the field at a non-governmental organization (NGO)

Participants did appreciate that Evidence Aid is accessible to a broad spectrum of people working in the humanitarian sector who have access to the internet. A mobile friendly app, which is not currently available, or the use of a responsive web design was suggested as a way to improve the overall user experience. Senior decision-makers highlighted the importance of having open-access resources and timely assessment data on current crises to inform decision-making, with one stakeholder stating:

“*There needs to be more open-access resources. Organizations need to share early on data from the field that would allow us to somehow get other actors to build the evidence to better inform our decisions.*”

A healthcare provider further emphasized the importance of open-access resources stating:

“*Open source access is still a big problem unless you have university library access.*”

### Credibility

Participants cited as a minor issue not clearly knowing what inclusion criteria are used to include best available evidence on the site. Participants emphasized that the direct and clear link to the Cochrane Library increased their level of trust of the evidence presented. For specific suggestions, participants wanted to see greater visibility given to major contributors and funders, with an advisor working at an NGO, stating:

“*Highlight the main funders of the site on front page to make it more transparent with emphasis on the major contributors to Evidence Aid.*”

### Value for the user

The lack of awareness among humanitarian aid workers about the existence of or value added by Evidence Aid was cited by participants as a minor issue. Several participants made comments about hearing of Evidence Aid but never using it because of lack of awareness about its value. An advisor and a field manager highlighted this during the interview, stating:

“*I heard of it but never used it. It has the potential of being super helpful. But not many people know about it now.*” – advisor working at an NGO

This prompted our participants to suggest that Evidence Aid should emphasize more clearly on their site why evidence matters in humanitarian action and to continue collaborating with other organizations to fill gaps with new systematic reviews.

## Discussion

Our study suggests that there are no ‘major’ problems (highest order) and only two ‘big’ problems (second highest order) that stakeholders’ experience with using Evidence Aid website, namely the lack of a search engine on the home page and that some full-text articles linked to or from the site are not accessible without payment to the publisher. Our study participants identified a positive feedback related to credibility (i.e. direct and clear link to the Cochrane Library increasing their level of trust of the evidence presented) that raises an important point that warrants highlighting. We found that users were inclined to make judgements about the trustworthiness of the Cochrane Library as the publishing source rather than critically assessing individual pieces of evidence, a similar finding in other studies [12]. Additionally, participants identified a minor issue related to value (i.e. the lack of awareness among humanitarian aid workers about the existence of or value added by Evidence Aid), that provides a key insight into the challenges with supporting the use of research evidence to inform decision-making in crisis zones, as highlighted in other studies [36–38].

Seven specific suggestions made by our participants and illustrated in Table 3 present actionable suggestions for improving Evidence Aid, many of which can also be applied to other evidence websites designed to support the use of research evidence in decision-making; these include (1) create a home page-based search engine; (2) strive to ensure that basic tasks can be easily accomplished on first use; (3) ensure that the search results are presented in a user-friendly way (e.g. turn the evidence available into explicit actionable points), in a language that can be read (i.e. in common first languages), and without jargon; (4) keep the site design simple, with images that are appropriate to crisis zones and capture users’ attention; (5) accommodate diverse user contexts (e.g. inability to pay for articles) and physical functioning (e.g. colour blindness); (6) ensure accuracy of the information on the site (e.g. correct years of publication); and (7) increase the value of Evidence Aid for the user by achieving the second part of the stated mission (i.e. enabling the use of evidence), whereby Evidence Aid or another group can choose from a variety of additional ways to enable the use of research evidence (e.g. rapid reviews).

A common challenge that stakeholders face when trying to use research evidence to inform their decision-making relates to the lack of knowledge management skills and infrastructure [6, 39–43]; for example, the huge volume of research evidence currently produced and scattered across journals, books, reports and websites, many of which require a payment to access. Evidence use includes not only the determination of what evidence is needed to inform a decision, but also how to best support the use of that evidence to its full potential. Our study found that Evidence Aid is contributing to strengthening efforts to support evidence-informed decision-making in crisis zones.

Our findings suggest the following three contributions to understanding evidence use in a crisis zone. First, many of our participants emphasized the need for evidence to be turned into explicit actionable points (e.g. check-lists). However, we recognize that this task is better delegated to a person or group that create connections between researchers and decision-makers (e.g. knowledge brokers). Second, our participants highlighted that evidence summaries must clearly indicate the basic findings from systematic reviews, including key messages that can be acted upon [10]. Third, our stakeholders raised the importance of having a well-organized website that consists of a wide variety of relevant information, allowing them easy and efficient access to the best available evidence in the limited time they have available to make, inform or advocate for a decision [11]. Clearly, stakeholders working in crisis zones have a diverse array of knowledge needs, and these findings reaffirm the importance of doing further scholarly work to better understand how to best support evidence use in crisis zones.

### Findings in relation to other studies

Our finding that participants did not identify any major problems (and only two big problems) with using Evidence Aid aligns with previous studies identifying that users generally find there are many helpful attributes of using evidence websites (e.g. multiple sources of information in one spot) [10, 15]. This study also aligns with other studies in putting forward specific suggestions to improve the use of evidence websites (e.g. functions in the users’ first language) [10]. Finally, this study complements existing literature in being the first study to specifically focus on an evidence website for crisis zones, elaborating on the information needs of stakeholders working in crisis zones and putting forward specific suggestions that address all facets of improving users experience; additionally, the research team is independent from Evidence Aid [4, 5, 10–12, 15, 35, 38, 44–47].

### Strengths and limitations

There are a number of strengths to this study. As far as we are aware, this is the first study to examine evidence website use in crisis zones and the first user-testing study to investigate the information needs of stakeholders working in crisis zones, which provides valuable insight on how best to meet their knowledge needs. Second, we interviewed a large number and diverse range of people for a study of this type, with the number higher than that thought to reveal 80% of known usability problems [28]. The diversity in our study lies within the types of stakeholders included, organizational affiliations, and whether the users employed Evidence Aid or not (and hence a likely broad sampling of the challenges stakeholders would face in navigating research evidence for use in crisis zones). Some notable differences in responses emerged across these diverse types of stakeholders and between users and non-users of Evidence Aid. However, there were no notable differences in responses across gender or participant ability to verbally communicate their insights in English. Finally, this study presents a non-affiliated examination of Evidence Aid, when there is a lack of third-party research about the effectiveness of evidence websites, with most existing research designed and conducted by groups associated with the website under study.

One potential limitation to this study is that all our interviews, except one, were conducted with stakeholders not physically present in a crisis zone at the time of the interview. Increased time pressure in crisis zones may influence participants’ views and experiences in finding relevant research evidence for decision-making. To mitigate this limitation, we purposively sampled participants who were either currently working or have worked within the last year in a crisis zone and we prompted them to consider real-life situations when responding.

### Implications for practice

There are four main implications, the first of which is that the developers of Evidence Aid should continue their efforts of providing the best available evidence on the effectiveness of humanitarian action while taking into account the specific suggestions, summarized above, to improve the site. These specific suggestions can also be applied to other evidence websites designed to support the use of research evidence in decision-making. Second, the developers of Evidence Aid site should consider whether they or another group are better positioned to fulfil the second part of their mission, namely ‘enabling the use of the best available research evidence’, by expanding their activities to include creating demand for research evidence, providing rapid reviews in response to decision-maker requests and institutionalizing the use of research evidence, among other options [48–52]. Third, senior decision-makers working in crisis zones should work with humanitarian aid workers to raise awareness of the existence of evidence websites, like Evidence Aid, and to build their capacity to find and use research evidence in decision-making. Finally, the users of Evidence Aid should continue to provide their feedback on how Evidence Aid and other evidence websites can best meet their knowledge needs.

### Future research

The next steps in research could be for researchers to explore stakeholders’ experiences with an updated version of Evidence Aid to ‘test’ (e.g. randomized controlled trials) if specific changes have improved the usability and use of the site. Additionally, researchers could evaluate future efforts by Evidence Aid or its partners to address the part of its mission focused on enabling the use of research evidence. Researchers could also explore other evidence websites (e.g. ReliefWeb, Cochrane database), which were most cited by our participants as their main source of obtaining information to find ways to adapt these websites’ strengths to improve Evidence Aid. Finally, researchers working in other domains (i.e. outside humanitarian crises) could use our methodology (i.e. diversity in user types of stakeholders and organizational affiliation) to explore stakeholders’ views and experiences with other evidence websites designed to support evidence-informed decision-making.

## Conclusion

Stakeholders in crisis zones found Evidence Aid to be useful, accessible and credible. However, they experienced some problems with the lack of a search engine on the home page and the fact that some full-text articles linked to or from the site require a payment. This is the first study to specifically focus on an evidence website for crisis zones, elaborated on the information needs of stakeholders, and put forward specific suggestions about how to improve evidence websites. By making evidence available, evidence websites provide one of the necessary inputs for evidence-informed decision-making processes. The absence of evidence websites creates a clear gap in supporting evidence-informed decision-making.

## Supplementary information

**Additional file 1..** Interview guide used in Test.

## Data Availability

All data generated or analysed during this study are included in this published article.
