# Supplementary material for: Stakeholders’ experiences with the evidence aid website to support ‘real-time’ use of research evidence to inform decision-making in crisis zones: a user testing study
Source: Health Res Policy Syst. 2019 Dec 30;17:106. doi: 10.1186/s12961-019-0498-y (PMC6936118; doi:10.1186/s12961-019-0498-y)
Supplement: Supplementary file 1 — Additional file 1.. Interview guide used in Test. [file 12961_2019_498_MOESM1_ESM.docx]

**Additional file 1:** Interview guide used in test

**Introduction**

Hello, my name is Firas Khalid and I want to thank you for taking your time to meet/speak with me today. My study is exploring your views and experiences of the Evidence Aid website. I would like to start this interview by asking some general questions. Please feel free to stop me at any point to ask for any clarifications.

- Denotes probes/prompts

**Section A: General Questions**

1. Do you have any questions for me before proceeding to the interview?
2. Could you please tell me more about the kind of work you are assigned to do?
3. Could you please describe some of your knowledge needs in relation to the line of work you do?

- Knowledge needs related to clinical management of patients in a crisis situation
- Knowledge needs related to operational logistical management (i.e., shelter, security, hygiene, mobile clinic set ups, mass immunizations, human resources issues, etc.,)?
- Knowledge needs related to policy-development in a crisis situation?

1. We define research evidence as the output of research that has been conducted in a systematic way and reported in a transparent manner. What kind of research evidence do you use to address your knowledge needs?

- Empirical (e.g., observational studies, surveys and case studies) and conceptual papers (e.g., theoretical papers)
- Primary or single studies and secondary research (e.g., systematic reviews and other forms of evidence synthesis)
- Indexed bibliographic databases or in what is called the grey literature
- Other types of information, including tacit knowledge or ordinary knowledge and stakeholder opinions

1. Where did you go to obtain research evidence to address your knowledge needs?

- Could you tell us about your online searching habits?
- What sources of online information do you usually use in relation to your work?

1. Had you heard about Evidence Aid before I contacted you for this interview?

❑ Yes ❑ No

1. Have you used Evidence Aid before?

❑ never used it before ❑ used it a few times ❑ used it a lot – please describe frequency

If the answer to #7 is used it a few times or used it a lot, proceed to section B of this interview guide: “user experience related task questions”

If the answer to #7 is never used it before, proceed to the following set of questions followed by section B of this interview guide

1. Why have you not used Evidence Aid Website before?

- Never heard of it
- Not relevant to my knowledge needs
- Could not locate it

1. Did you use any other evidence websites before?

❑ **YES**  ❑ **NO**

1. If **YES,** could you please state which ones?

**Section B: “user experience related task questions”**

Participants now will be asked to sit in front of a computer. Participants will be asked to think out loud during the whole session and reminded of this throughout the interview.

# Could you please now navigate to Evidence Aid website:

# What are your initial reactions to the front page?

# Could you please now find a relevant review on non-communicable diseases in humanitarian crisis?

# Could you please now find a relevant review on epidemic outbreaks in humanitarian crisis?

# Could you please now find a relevant review on camp coordination in humanitarian crisis?

# Could you please now find a relevant review on displaced people in humanitarian crisis?

# Could you please now look for specific content related to a topic that is relevant to your field or professional interests?

# Could you please now look for a relevant systematic review related to a topic that is relevant to your field or professional interests?

# Could you please now look for evidence-based guidelines related to a topic that is relevant to your field or professional interests?

# Could you please now look for a user-friendly summary of reviews related to a topic that is relevant to your field or professional interests?

# Where would you go if you run into a problem and need help to navigate the site?

# Could you please now start a new search?

# Where would you go if you wanted to know more about who is behind the site, how it was developed, target audience, and key advantages of using the site?

# Any major problems you faced with the tasks we asked you to perform?

# Any big problems or frustrations you faced while performing the tasks?

# Any minor issues you would like to highlight?

# Any positive feedback you would like to provide?

# Any specific suggestions for improving your experience?

**Section C: Questions related to overall experience**

1. Findability: To what extent did you find Evidence Aid navigable where you can easily locate and find what you needed?
2. Usability: To what extent did you find it easy to use and satisfying to use Evidence Aid website?
3. Usefulness: To what extent did you find that Evidence Aid fills a gap that you needed filled?
4. Desirability: Could you describe how you found Evidence Aid use of images, identity, brand, and other elements of the design that you found to be desirable?
5. Accessibility: To what extent did you find that Evidence Aid easily available to you and other decision-makers’ like you or to ones that have difference preferences or disabilities?
6. Credibility: To what extent did you trust and believe what is presented to you on Evidence Aid and what elements of Evidence Aid website influenced this trust?
7. Value: To what extent did you find Evidence Aid website advancing the mission of the Evidence Aid group of ““providing the best available evidence on the effectiveness of humanitarian action and enabling its use”?
8. Is there anything else that you would like to add?

**Closing remarks:**

1. Do you know one or two others who would be well suited to participate in a similar interview.
2. Finally, we will be analyzing the information you and others give us. We will be sharing the results of our study with you at a later date. In the meantime, thank you for your time.
